# INTEREST GROUP SESSION—INDIGENOUS PEOPLES: REVEALING THE HEALTHY BRAIN INITIATIVE’S ROAD MAP FOR INDIAN COUNTRY: OPPORTUNITIES FOR ENGAGEMENT

**DOI:** 10.1093/geroni/igz038.1334

**Published:** 2019-11-08

**Authors:** Heidi Holt, Blythe S Winchester

**Affiliations:** 1 Centers for Disease Control and Prevention, Atlanta, Georgia, United States; 2 Cherokee Indian Hospital, Cherokee, North Carolina, United States

## Abstract

This landmark document, The Healthy Brain Initiative: Road Map for Indian Country, is the first-ever public health guide focused on dementia in American Indian/Alaska Native (AI/AN) communities. It is intended as a tool for leaders of the 573 federally recognized Indian tribes, as well as state-recognized tribes, to engage their communities in this important issue. Early in the development of the HBI Public Health Road Map for Dementia, CDC recognized strategies that may work for state and local public health agencies likely would need to be tailored by native communities due to culture and unique contexts. This Road Map for Indian Country (Road Map) can be used to support a dialogue within a Native community about how to promote wellness across the lifespan and best support older adults with dementia, their families, and caregivers. The panel will consist of 5 presenters and 1 discussant. Bill Benson, International Association of Indigenous Aging, will discuss the background and need for the project. Molly French, the Alzheimer’s Association, will describe the process used to create the Road Map. Heidi Holt, CDC, will describe the content and goals of the Road Map. Kelsey Donnellan, Association for State and Territorial Health Officials (ASTHO), will present key Indian Country products and Messages that accompany the Road Map. Lisa McGuire will present relevant Behavioral Risk Factor Data, including data specific to the AI/AN population. The discussant will wrap up the panel and provide unique insights as to the use and future for this important document.

